# Supplementary material for: Uncovering changes in microbiome profiles across commercial and backyard poultry farming systems
Source: Microbiol Spectr. 2023 Aug 21;11(5):e01682-23. doi: 10.1128/spectrum.01682-23 (PMC10580917; doi:10.1128/spectrum.01682-23)

Supplementary Figure 1: Alpha diversity (Chao 1) measurement of fecal samples of backyard and commercial farms combined. V1, V2, and V3 are first, second, third visits respectively.

The figure below illustrates the alpha diversity measurement (Chao1) of fecal samples collected from broiler chickens during multiple visits. The dataset combined samples obtained from both commercial and backyard farms. The data clearly demonstrates a progressive increase in the alpha diversity measurement with each subsequent visit.

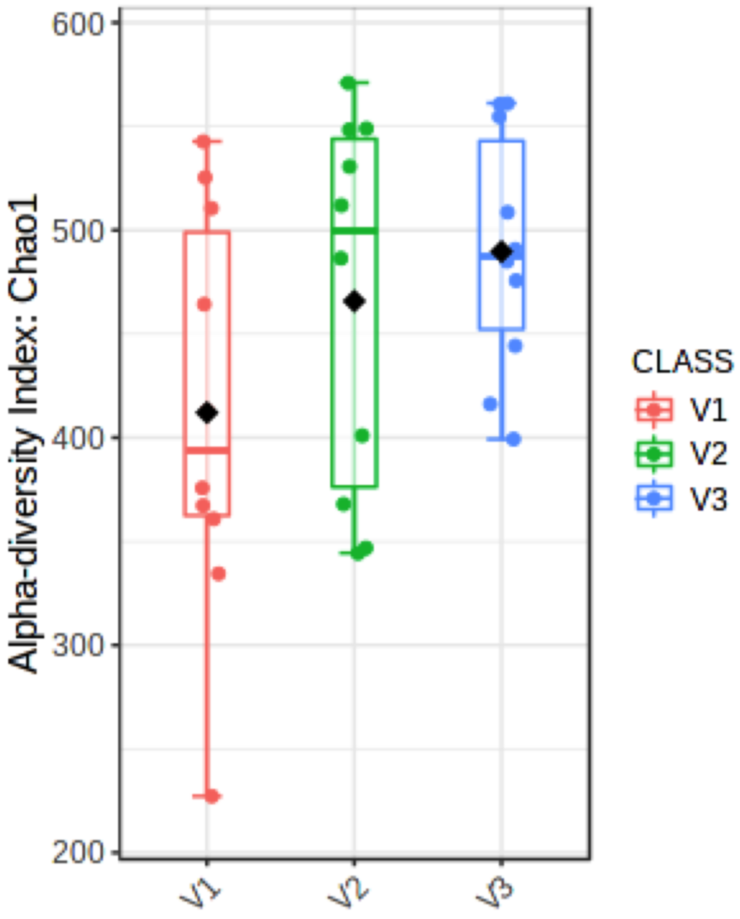

Supplement: Figure S1 — Alpha diversity measurement (Chao1) of fecal samples collected from broiler chickens during multiple visits. The data set combined samples obtained from both commercial and backyard farms. The data clearly demonstrate a progressive increase in the alpha diversity measurement with each subsequent visit. [file spectrum.01682-23-s0001.pdf]
